# The Most Promising Biomarkers of Allogeneic Kidney Transplant Rejection

**DOI:** 10.1155/2022/6572338

**Published:** 2022-05-28

**Authors:** Karolina Rogulska, Iwona Wojciechowska-Koszko, Barbara Dołęgowska, Ewa Kwiatkowska, Paulina Roszkowska, Patrycja Kapczuk, Danuta Kosik-Bogacka

**Affiliations:** ^1^Department of Microbiology, Immunology and Laboratory Medicine, Pomeranian Medical University, Powstańców Wlkp. 72, 70-111 Szczecin, Poland; ^2^Department of Nephrology, Transplantology and Internal Medicine, Pomeranian Medical University, Powstańców Wlkp. 72, 70-111 Szczecin, Poland; ^3^Department of Biochemistry and Medical Chemistry, Pomeranian Medical University, Powstańców Wlkp. 72, 70-111 Szczecin, Poland; ^4^Independent Laboratory of Pharmaceutical Botany, Pomeranian Medical University, Powstańców Wlkp. 72, 70-111 Szczecin, Poland

## Abstract

Clinical transplantology is a constantly evolving field of medicine. Kidney transplantation has become standard clinical practice, and it has a significant impact on reducing mortality and improving the quality of life of patients. Allogenic transplantation induces an immune response, which may lead to the rejection of the transplanted organ. The gold standard for evaluating rejection of the transplanted kidney by the recipient's organism is a biopsy of this organ. However, due to the high invasiveness of this procedure, alternative diagnostic methods are being sought. Therefore, the biomarkers may play an essential predictive role in transplant rejection. A review of the most promising biomarkers for early diagnosis and prognosis prediction of allogenic kidney transplant rejection summarizes novel data on neutrophil gelatinase-associated lipocalin (NGAL), kidney injury molecule-1 (KIM-1), C-X-C motif chemokine 10 (CXCL-10), cystatin C (CysC), osteopontin (OPN), and clusterin (CLU) and analyses the dynamics of changes of the biomarkers mentioned above in kidney diseases and the mechanism of rejection of the transplanted kidney.

## 1. Introduction

Clinical transplantology is a constantly evolving field of medicine [1]. Developments in surgical techniques and changes in immunosuppressive therapy have made effective organ and tissue transplantation possible [2]. Currently, in addition to performing traditional organ transplants, experimental head and face transplants are being attempted [3, 4].

Kidney transplantation has become standard clinical practice over the past few decades [5]. It has a significant impact on reducing mortality and improving patients' quality of life by not requiring haemodialysis or peritoneal dialysis [6]. The number of transplantation procedures performed globally, including kidney transplantation, increases every year. However, the rejection of transplanted organs and tissues is a significant problem. To date, the mechanisms that allow long-term functional maintenance of the transplanted kidney have not been thoroughly understood.

The monitoring of transplanted kidney is based on physical examination, urine volume, the assessment of albuminuria or proteinuria, serum creatinine, and glomerular filtration rate (GFR) estimation based on serum creatinine [7]. Serum creatinine levels are the most commonly used biochemical parameter; they increase late in injury and are nonspecific for the type of injury [8]. It is thought that it is likely to be a factor in determining long-term graft survival [9]. However, serum concentrations of this parameter are not sensitive and specific for estimating the condition of the graft [10]. Additionally, the serum creatinine level is not able to predict or evaluate the progression of chronic injury and as a consequence is not specific or predictive [8]. The histological examination through renal biopsy remains the gold standard for diagnosis to evaluate the rejection process of the transplanted kidney, which can indicate chronic immune injury or display interstitial fibrosis and tubular atrophy (IFTA) [11]. The biopsies are associated with sampling error, and there is a lack of consensus around both histologic interpretation and the effectiveness of treatment [12]. This method has several drawbacks, characterised by low sensitivity, low specificity due to heterogeneity of processes underlying the same lesion, lack of standardization and of quantitative thresholds, and sampling errors [13]. Due to the high invasiveness of this procedure, alternative methods of diagnosis are being sought [14, 15]. The evaluation of kidney transplant rejection also used the imaging techniques, including monitor renal graft perfusion using Doppler ultrasound, contrast-enhanced ultrasound (CEUS), and magnetic resonance imaging (MRI) as well as nuclear imaging [16]. Attempts are now being made to minimise rejection rates by monitoring anti-HLA antibody titres and introducing new biomarkers, recently for potential use in clinical practice, including measurement of serum donor-derived cell.

In this review, we described the most promising biomarkers for early diagnosis and prognosis prediction of allogenic kidney transplant rejection. This review is based on the scientific articles found in validated sources such as PubMed and the National Centre for Biotechnology Information (NCBI). The keywords used were biomarkers AND kidney AND transplant AND rejection OR neutrophil gelatinase-associated lipocalin OR lipocalin-2 OR kidney injury molecule-1 OR hepatitis A virus cellular receptor 1 OR T-cell immunoglobulin mucin receptor 1 OR C–X–C motif chemokine 10 OR interferon-*γ*-inducible protein-10 OR cystatin C OR osteopontin OR clusterin. Inclusion criteria were as follows: human studies that used noninvasive methods for assessing biomarker, in vitro animal studies, publication in a peer-reviewed journal, review articles and research article, and articles in languages other than English. Exclusion criteria were as follows: gray papers (e.g., conference proceedings and abstracts), case reports, short communication, and books. Following the application of these criteria, 205 papers were selected for review.

## 2. Biomarkers of Allogeneic Kidney Transplant Rejection

A biomarker is a characteristic that is objectively measured and evaluated as an indicator of a normal biological process, pathogenic process, or pharmacological response to a therapeutic intervention [17]. Biomarkers are used for (1) diagnosis of patients with a disease or an abnormal organ function, (2) severity of disease, (3) prognosis of a disease, and (4) monitoring of a response to a medical procedure [18]. Biomarkers can be classified into seven types based on their purpose, as follows: susceptibility or risk, diagnostic, prognostic, predictive, monitoring, pharmacodynamic/response, and safety biomarkers [13]. The role of risk biomarkers is to identify patients with a high probability of developing the disease before clinical symptoms appear. Prognostic biomarkers are aimed at identifying patients who require treatment and patients who have the potential to stop disease progression. In contrast, a predictive marker helps to determine the type of treatment needed to stop disease progression. Monitoring markers are used to assess disease activity. The dynamics of drug action in the body are assessed by pharmacodynamic markers. Safety biomarkers are used to assess the toxicity of the treatment administered [8]. In addition, they allow the assessment of the dynamics of immunological changes and thus predict the body's response to a transplant [19, 20] (Table 1).

**Scheme 1:**
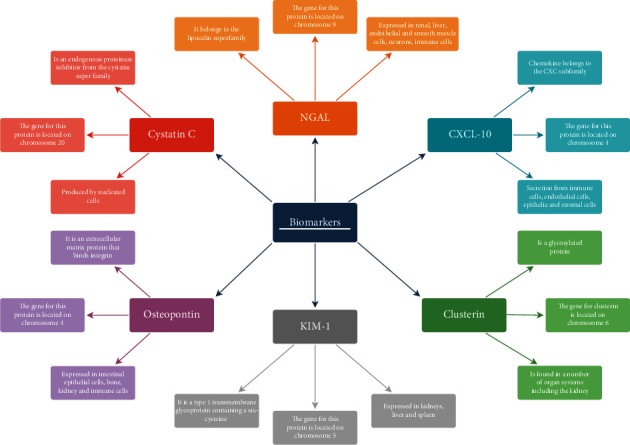
Schematic of key features of biomarkers of kidney transplant rejection.

We herein reviewed the current literature on potential biomarkers: neutrophil gelatinase-associated lipocalin (NGAL), kidney injury molecule-1 (KIM-1), C-X-C motif chemokine 10 (CXCL-10), cystatin C (CysC), osteopontin (OPN), and clusterin (CLU) [32–34] (Scheme 1, Table 2). Additionally, the pros and cons of the mentioned biomarkers are presented in Table 3.

### 2.1. Neutrophil Gelatinase-Associated Lipocalin (NGAL)

Neutrophil gelatinase-associated lipocalin is known as lipocalin-2, 24p3, siderocalin, or uterocalin which is a 21 kD protein of the lipocalin superfamily [49]. It is found in 3 isoforms: monomeric (25 kDa) or dimeric (45 kDa), and only a small fraction is heterodimeric (135 kDa—complexed with gelatinase) [50]. NGAL is synthesised during a narrow window of granulocyte maturation in the bone marrow but also may be induced in epithelial cells in the setting of inflammation or malignancy [51, 52]. The gene for this protein is located on chromosome 9 [53].

NGAL is expressed in renal, liver, endothelial, and smooth muscle cells, neurons, and immune cells, including macrophages and dendritic cells [54, 55]. They are secreted by neutrophils in inflammatory conditions and act as acute-phase proteins [56]. The plasma concentration of NGAL (sNGAL) is approximately 70 ng/ml in healthy humans [57]. NGAL levels can also be measured in urine (uNGAL) [58]. The reference range of uNGAL is the subject of many studies. According to Lima et al. [59], it ranges from <9 to 54.5 ng/ml. NGAL is considered to be a marker of acute tubular cell injury. The primary ligands for NGAL are siderophores and metalloproteinase 9 (MMP-9) [60]. Siderophores are molecules with the ability to bind and transport iron. They are found in many living organisms, including bacteria [61]. NGAL maintains bacteriostasis by binding bacterial siderophores and restricting their growth [62]. In contrast, MMP-9 is a protein that degrades the extracellular matrix (ECM). It leads to the formation of intercellular spaces and altered activity of substances, including chemokines, cytokines, and growth factors that play essential roles in carcinogenesis [63]. The complex of NGAL with metalloproteinase 9 enhances its proteolytic activity while inhibiting the inhibitor TIMP-1. It results in an increase in local and distant tumour cell production [64]. Lipocalin-2 may therefore serve as an adverse prognostic factor in cancer patients [65]. NGAL enhances the action of MMP-9 also in cardiovascular disease. Excessive metalloproteinase activity may lead to thrombosis by increasing atherosclerotic plaque instability [66].

Under physiological conditions, lipocalin-2 undergoes glomerular filtration and reabsorption in proximal renal tubules [67]. Under physiological conditions, NGAL expression remains low but increases responding to epithelial cell injury [68]. Therefore, the amount of lipocalin-2 in urine may originate from damaged first-order tubules and impaired clearance of this protein. However, it appears that structures not involved in NGAL excretion can also induce the production of this lipocalin as a result of damage. Therefore, the urinary fraction of NGAL is mainly the result of synthesis in the kidney [49]. The plasma concentration of neutrophil gelatinase-associated lipocalin results from tissue production of this protein responding to injury. An example of this phenomenon is acute kidney injury (AKI), which progresses with the destruction of other organs—lungs and liver. In patients with AKI, a common complication is the development of respiratory failure. The prognosis of patients suffering from both conditions is the worst among coexisting AKI with other diseases [69]. Another complication may be liver failure or cirrhosis, which also have a poor prognosis [70]. Additionally, NGAL can be released by neutrophil granulocytes as an acute-phase protein. Following AKI, the glomerular filtration rate (GFR) is also reduced, increasing NGAL [71]. NGAL levels in patients with AKI increase up in blood and urine to 300-fold (0.1–30 *μ*g/ml) and 1000-fold (0.04–40 mg/ml), respectively [18, 72]. A meta-analysis of 52 research articles involving a total of 13,040 patients concluded that determination of both sNGAL and uNGAL levels could capture individuals at high risk of developing AKI [73].

NGAL may be an important marker of kidney injury. Compared to creatinine, whose concentration increases several hours after renal cell destruction, its increase in both urine and plasma can be observed after only about 2 hours [74]. The potential role of this protein in the monitoring process of renal transplant patients is also currently being studied. NGAL can be used to assess transplant status as early as a few hours after transplantation. Delayed transplant function (DGF) is a disorder that occurs due to reperfusion abnormalities in the organ after surgery. It develops in approximately 25% of kidney recipients [35]. A common complication in patients who develop DGF is transplant loss a year or two after transplantation [36]. Kanter et al. [21] found that uNGAL in renal transplant recipients in the first days after transplantation was lower in patients without reperfusion injury. In addition, they showed that falling levels of this protein on day 3 after surgery were a good predictor of renal function one month after transplantation. Capelli et al. [22], based on retrospectively evaluating the clinical and laboratory data of 72 patients after renal transplantation, concluded that uNGAL combined with other markers could be more helpful in the early evaluation of renal function in the first week following kidney transplantation. Additionally, Lacquaniti et al. [23] found that both urinary and serum NGAL levels provide reliable information for predicting kidney injury. According to Rostami et al. [37], NGAL may be a biomarker for AKI following kidney transplantation. In a prospective cohort study, in 64 adults who underwent kidney transplantation, the authors found that uNGAL level was more remarkable in recipients with AKI than patients who had no AKI. Its increase was observed in recipients at 2 hours after surgery. In a study on an Iranian population (*n* = 37), Pezeshgi et al. [25] confirmed the usefulness of sNGAL in diagnosing AKI and demonstrated its essential role in diagnosing AGF—acute kidney injury in transplant patients. However, Rahimzadeh et al. [26] demonstrated that serum and urinary NGAL levels within the first week after renal transplantation in children (*n* = 27) could be induced by injury and drugs, including bisphosphonates, cephalosporin, and cisplatin. They also found that the lack of standardisation of lipocalin-2 measurement was a major problem in interpreting the results. On the other hand, Field et al. [20] noted a significant increase in sNGAL levels on day 1 in patients undergoing HLA-incompatible renal transplantation (*n* = 94), in whom the development of rejection occurred within one month. The specificity and sensitivity of this marker were approximately 60-70%. A similar relationship was noted by Kohei et al. [27]. They studied twelve patients clinically diagnosed with acute rejection by renal biopsy. They highlighted that uNGAL was the most sensitive of these markers to detect acute kidney allotransplant dysfunction after living-donor kidney transplantation. Furthermore, they observed that creatinine levels are not sensitive and specific enough to be a useful biomarker in the postoperative period. Heyne et al. [24], studying uNGAL in 182 outpatient renal allotransplant recipients on maintenance immunosuppression, noted that determination of urinary NGAL levels could be a parameter to differentiate acute allotransplant rejection from other causes of AKI in follow-up after kidney transplantation. Kielar et al. [38], performing a study on 109 kidney recipients with stable transplant function one year after transplantation, found that uNGAL and sNGAL and NGAL/creatinine can be used to estimate the change in kidney transplant function.

### 2.2. Kidney Injury Molecule-1 (KIM-1)

Kidney injury molecule-1 is also named hepatitis A virus receptor (HAVCR1) and T cell immunoglobulin mucin receptor 1 (TIM-1) [75]. It is a type 1 transmembrane glycoprotein containing a six-cysteine immunoglobulin-like domain and a mucin domain in its extracellular region [76, 77]. KIM-1/HAVCR/TIM-1 is a protein of approximately 104 kD [78]. KIM-1 plays different roles in T and B cell biology [79]. The gene for this protein is located on chromosome 5q33.2 [80]. KIM-1 is expressed in the kidney, liver, and spleen [81]. Healthy kidney tissue expresses very low or undetectable levels of KIM-1. In addition, this protein is also undetectable in urine [82, 83]. Studies have shown that KIM-1 plays different roles via various molecular targets in immune diseases and kidney injury [81]. KIM-1 is expressed on the apical membrane surface of proximal tubular epithelial cells of the kidney, especially in the S3 segment, responding to hypoxia or renal tubular injury [84]. Its extracellular domain is detached by metalloproteinases and secreted into the urine. This extracellular ectodomain of KIM-1 is a quantitative marker of kidney injury [85]

KIM-1 downregulates proximal tubular cell cytokine secretion, downregulation of translational changes through nuclear factor kappa-light-chain-enhancer of activated B cells (NF-*κ*B) pathway, and interaction with phosphatidylinositol3 PI3 kinase subunit p85 [86].

In this mechanism, the extracellular part of KIM-1 is detached from the rest of the glycoprotein via proteins of the metalloproteinase family and transported into the urine [87]. Therefore, it is believed that KIM-1 can be used to diagnose kidney disease [88]. According to the European Medicines Agency and the US Food and Drug Administration, this protein has been recognised as a biomarker of kidney damage following nephrotoxic drugs [83, 89].

Like NGAL, KIM-1 appears in urine after approximately 24-48 hours of damage to various nephrotoxic factors induced [90]. The concentration of this glycoprotein may increase before significant changes in estimated glomerular filtration rate (eGFR) occur and thus foreshadow kidney damage [91]. Additionally, Nowak et al. [92] demonstrated that plasma KIM-1 (pKIM-1) is a good predictor of impaired renal function in nonproteinuric patients with type 1 diabetes. Gohda et al. [93] studying this biomarker in patients with type 2 diabetes (*n* = 602) found that serum KIM-1 (sKIM-1) correlates more strongly with eGFR levels than urinary KIM-1 (uKIM-1). Ren et al. [94] noted that uKIM-1 levels might be used as early, sensitive indicators of AKI in patients with burns of varying degrees. Based on a review of publications from 2000 to 2007 on the reliability of serum and urinary biomarkers in human subjects when used for the diagnosis of established AKI or early AKI, it was concluded that uKIM-1 could be used for the differential diagnosis of renal tubular necrosis and other conditions causing renal destruction [95]. Alderson et al. [96] observed that, in patients with chronic kidney disease (CDC), pKIM-1 are independent risk factors for progression to end-stage renal disease (ESRD). In the case of AKI, KIM-1 has been found to exhibit prophagocytic actions, resulting in the destruction of inflammatory cells and reducing the focus of inflammation [81]. The opposite is true for CKD, as this protein increases inflammation and apoptosis of renal cells. Schulz et al. [97] on a Swedish population (*n* = 4739) for over 16 years found that KIM-1 correlates with a decrease in eGFR and risk of chronic kidney disease. However, Sinkala et al. [98] studying patients with acute kidney injury or CDC found that KIM-1 is not a promising biomarker for the diagnosis of kidney disease, compared to the standardly measured parameters creatinine and urea, which are the best indicators of organ failure because their accuracy increases as transplant function deteriorates.

Kidney injury molecule-1 is also an important marker of kidney transplant rejection [99]. Jin et al. [39] studied sKIM-1 and osteopontin (OPN) in patients who were classified into acute rejection group (*n* = 32), nonrejection group (*n* = 45), and healthy controls (*n* = 78). The authors concluded that sKIM-1 might be a marker for the prediction of early kidney transplant rejection. In addition, they observed that concurrent sKIM-1 and OPN significantly increased the efficiency of predicting this process. Similarly, Shabaz et al. [28] studied uKIM-1 mRNA expression and urinary and serum KIM-1 proteins in renal allotransplant recipients diagnosed with acute allotransplant rejection (*n* = 24) and chronic allotransplant dysfunction (*n* = 19) and patients with well-functioning transplants (*n* = 42). They concluded that KIM-1 could be used to monitor renal transplant recipients, which may contribute to earlier diagnosis of organ rejection, mainly of the acute type and chronic transplant dysfunction. In contrast, van Timmeren et al. [40], in a study of renal transplant recipients (*n* = 145), showed that urinary excretion of KIM-1 is an independent factor for transplant loss in the recipient more than 12 months after surgery.

### 2.3. C-X-C Motif Chemokine 10 (CXCL-10)

CXCL-10, also known as interferon-*γ*-inducible protein-10 (IP-10), is a chemokine belonging to the CXC subfamily [100]. There are four subfamilies of chemokines: CXC, CC, C, and CX3C [101]. CXC is composed of two cysteines located at the N-terminus separated by a single amino acid that can be variable, which distinguishes it from the other chemokine subfamilies where these amino acids are located next to each other [102]. Chemokines are generally small molecules between 7 and 15 kD [103]. The gene for this protein is located on chromosome 4. Secretion of CXCL-10 from leukocytes, neutrophils, eosinophils, monocytes, epithelial, endothelial, and stromal cells, and keratinocytes occurs responding to several proinflammatory factors, most notably interferon-*γ* (IFN-*γ*) [104, 105]. It modulates angiogenesis in conditions including wound healing, ischemia, and neoplasia [106]. CXCL-10 is secreted by leukocytes in the kidney transplant and is an inflammation marker. Schaub et al. [107] demonstrated that the sensitivity and specificity of urinary CXCL-10 (uCXCL-10) exceeded those of creatinine concentrations in serum. CXCL-10 acts by activating CXC-receptor 3 chemokines found on the surface of some cells—NK cells, helper T cells, macrophages, and dendritic cells [104]. The primary function of CXCL-10 is to participate in chemotaxis [108]. It is also involved in forming diseases, including Graves-Basedow or autoimmune thyroiditis [109]. CXCL-10 has a strong influence on the occurrence of cardiovascular lesions, including coronary syndromes and atherosclerosis [110, 111]. Chemokines of the CXC subfamily have also been shown to have pro- or antiangiogenic effects, resulting in tumour formation, mainly melanoma [112]. CXCL-10 is an inhibitor of angiogenesis and therefore has anticancer effects [113].

Due to the role of CXCL-10 in the body, it has been found that it can be used for the noninvasive diagnosis of kidney disease. Watson et al. [114] observed that levels of this chemokine could help diagnose early acute kidney injury in patients, including those caused by immune-independent factors. According to Marie et al. [115], it is a very sensitive marker in detecting nephritis during systemic lupus erythematosus (SLE). In addition, Reyes-Thomas et al. [116] believe that this chemokine is helpful in monitoring treatment in patients with SLE. The role of CXCL-10 in allogeneic kidney transplant rejection has been the subject of many studies worldwide [117]. Ciftci et al. [29], investigating living-related donor renal transplant recipients, showed that uCXCL-10 is well identified in patients with an acute cellular type of kidney rejection and correlates with plasma creatinine levels. In contrast, Rabant et al. [10], based on the results of a highly sensitised cohort of 244 renal allotransplant recipients, concluded that monitoring urinary CXCL-10 and creatinine levels and then calculating the ratio of these two parameters can effectively determine the risk of antibody-dependent transplant rejection. Blydt-Hansen et al. [118] noted that the ratio of CXCL-10 to creatinine in children is a promising biomarker of acute cellular rejection. According to Matz et al. [41], CXCL-10 chemokine levels may predict the development of acute cell-type rejection. These findings predate the renal biopsy image by several days. Determination of CXCL-10 mRNA in urine, according to Tatapudi et al. [119], is an ideal biomarker of rejection and shows 100% sensitivity as confirmed by biopsy. Rotondi et al. [42] tested for CXCL-10 pretransplantation sera from 316 cadaver kidney transplant recipients (*n* = 316). The authors demonstrated that it would be appropriate to determine the levels of this chemokine before transplantation, as high pretransplant serum CXCL-10 levels may indicate a high risk of severe rejection and transplant failure. Similar conclusions were reached by Lazzeri et al. [43], studying serum CXCL-10 levels of 316 cadaveric kidney-transplant recipients (*n* = 316). They demonstrated that pretransplant serum CXCL-10 levels greater than 150 pg/ml predispose to severe transplant rejection. Jackson et al.'s [44] analysis of adult and paediatric transplant recipients found that urine CXCL-10 levels can increase in acute transplant rejection and BK virus infection. Still, this chemokine cannot differentiate between these conditions. Weseslindtner et al. [120], studying a group of 85 kidney recipients, found that CXCL-10 levels can increase with BK virus replication and the onset of nephropathy during infection with this pathogen.

### 2.4. Cystatin C (CysC)

Cystatin C is an endogenous proteinase inhibitor (~13.4 kD) from the cystatin superfamily of cysteine protease inhibitors inhibiting mainly cathepsins L, B, and H [121, 122]. It is composed of 120 amino acids forming a polypeptide chain [123]. The gene for this protein is located on chromosome 20 [124]. CysC plays a vital role in the intracellular catabolism of proteins and peptides. It is produced by nucleated cells at a constant level and is present in all body fluids in the body [125, 126]. The reference range in healthy individuals should be between 0.72 and 1.06 mg/l [127]. Serum concentrations appear to be independent of sex, age, and muscle mass. CysC concentrations may be altered in patients with thyroid disease and those taking high doses of corticosteroids [128, 129]. This protein is freely filtered in the glomeruli, undergoes reflux reabsorption, and is catabolised in the renal tubules [130]. In healthy individuals, essentially, no CysC is excreted in the urine [131]. When the renal tubules are damaged, these processes are disrupted, and cystatin appears in the urine [132]. The half-life of CysC is 1.5 hours [133]. Unlike creatinine, the concentration of CysC in the body does not depend on gender, age, or muscle mass [134]. Therefore, serum CysC is considered by many researchers as a better marker for estimating the dynamics of GFR changes than creatinine [135]. Villa et al. [136] observed that serum CysC is a better marker of GFR than creatinine in unstable patients with acute renal failure. Zheng et al. [137] studying a group of 425 patients with chronic hepatitis B found that cystatin C may be an essential indicator of developing renal functional impairment in these patients. The concentration of this protein is also a good factor for diagnosing AKI, rising before changes in creatinine [138]. It is estimated to precede the rise in creatinine levels by two days in patients at an advanced stage of kidney damage [139]. Soto et al. [140], in a cohort study in which they examined serum and urinary CysC in a heterogeneous group of patients (*n* = 616) presenting to a tertiary care emergency department, showed that serum CysC testing allows the diagnosis of AKI but has no value as a marker to differentiate between AKI and chronic kidney disease. Additionally, Briguori et al. [141], studying consecutive patients with CDC undergoing either coronary and/or peripheral angiography and/or angioplasty (*n* = 410), demonstrated that CysC could serve for early diagnosis and prognosis of the contrast-induced acute kidney. Patel et al. [142], studying patients with chronic pancreatitis, neoplasm, chronic liver disease, and chronic kidney disease, found an increase in baseline serum CysC was associated with AKI in patients with acute pancreatitis. Tarif et al. [143], studying patients with acute renal failure (*n* = 73) and control subjects (*n* = 300), found that creatinine and serum CysC are a good marker of renal function in acute renal failure patients especially those with worsening renal function. Based on electronic databases, Nakhjawan-Shahraki et al. [144] noted that CysC is a sufficient predictor for detecting AKI in children. The study by Safdar et al. [145] confirmed that cystatin C is a sensitive marker in very severe acute kidney injury in children, but only when its concentration is determined within 24 hours of the start of hospitalisation. A similar value of CysC in AKI diagnosis was discovered by Lagos-Arevalo et al. [146], conducting a study on a 150-person group of children admitted to the intensive care unit.

Cystatin C is also a marker of allogeneic kidney rejection. Krishnamurthy et al. [45] concluded that CysC as an additional diagnostic parameter in assessing transplanted organ function might be helpful and serve to tailor immunosuppressive treatment. Changes in GFR, which are a consequence of deteriorating transplant function and thus an increased risk of rejection, according to Taghizadeh-Afshari et al. [30], can be detected by the determination of cystatin C, which at 14 days posttransplantation exceeds the sensitivity and specificity of creatinine. Similar conclusions were reached by Le Bricon et al. [147], considering CysC to be a more accurate marker than creatinine and further positing a role for this protein in assessing the toxic effects of treatment.

### 2.5. Osteopontin (OPN)

Osteopontin, also referred to as bone sialoprotein 1 (BSP-1), secreted phosphoprotein 1 (SPP1) and early T lymphocyte activation 1 (ETA-1) [148]. It is an extracellular matrix protein (~35 kD) built from a polypeptide chain 314 amino acids long, containing an arginine-glycine-asparagine sequence that binds integrin [149, 150]. Osteopontin is encoded by a single-copy gene located on the human chromosome 4 (4q13) [151]. OPN expression is observed in various tissues and cells, including intestinal epithelial cells, bone, kidney, and immune cells, such as macrophages, dendritic cells, and T lymphocytes [152, 153]. In healthy subjects, the serum osteopontin concentration should be around 23.56 ng/ml [154]. OPN is involved in various physiological and pathophysiological processes, including tissue and bone remodelling, inflammation, cell survival atherosclerosis, and kidney damage [155–158]. Its principal function is to bind osteoclasts to bone [159]. In addition, it influences the regulation of the immune system, acting on a principle similar to that of cytokines [160]. It plays a significant role in the development of chronic inflammatory diseases [161]. It may also contribute to the development of cancer [162]. In the kidney, osteopontin is produced in the distal part of the nephron [163]. It likely contributes to vessels' formation in the kidney [164]. OPN is thought to reduce kidney stone formation [165]. Lorenzen et al. [166] found that OPN inhibitors could be used as a novel therapeutic target of albuminuria. Studies have also shown the involvement of OPNs in the formation of renal lesion characteristic of diabetic nephropathy [167]. According to Wong et al. [168], increased expression of this protein is a predictive factor in bladder cancer. In addition, Sim et al. [169] consider that OPN together with carbonic anhydrase IX and C-reactive protein is a promising biomarker in renal cell carcinoma. Feldreich et al. [170] showed that osteopontin plays a vital role in both cardiovascular and kidney diseases. Higher urinary OPN can predict deterioration of kidney function in the CDC, while OPN can estimate the risk of cardiovascular death based on plasma. In patients with acute kidney injury, Lorenzen et al. [171] observed an increase in OPN levels, which was a predictor of mortality from this disease at four weeks in severely ill patients. Askenazi et al. [172] showed that urinary OPN is also a promising biomarker for detecting AKI in neonates, similar to NGAL and KIM-1. Varalakshmi et al. [173] showed that plasma osteopontin can correlate with disease severity in a group of 35 AKI patients with renal replacement therapy.

Osteopontin also appears to be a promising biomarker in kidney transplant rejection due to its essential role in the inflammatory process [174]. Rouschop et al. [175] observed an increase in tubular expression of OPN (the ligands of CD44) in recipients, which was confirmed by biopsy results. In addition, they found that this protein may be involved in the development of renal rejection by enhancing the influx of monocytes. Alchi et al. [48] examined renal biopsies from patients with acute rejection, protocol biopsies without rejection, and perioperative donor biopsies for intrarenal expression of OPN. They demonstrated increased levels of this protein in biopsies from renal allotransplants with acute rejection. Wang et al. [31] consider that OPN levels in body fluids, especially plasma, predict and evaluate ACR severity in renal transplant recipients. The diagnostic findings coincided with the changes seen in the image of the biopsy taken at the same time. According to Zhao et al. [46], OPN levels may also increase in the mechanism of graft-versus-host disease when donor immune cells attack the recipient organism.

### 2.6. Clusterin (CLU)

Clusterin, also known as apolipoprotein J (CLU), is a glycosylated protein composed of two chains, *α* and *β*, linked by disulfide bonds [176]. In humans, it occurs in two isoforms. The secretory type, with a mass of about 80 kD, has the task of removing residues formed after apoptosis and the nuclear type with a mass of 50 kD is responsible for DNA repair [177]. The clusterin gene is located on chromosome 8 [178]. CLU is involved in both apoptotic and antiapoptotic pathways and is found in some organ systems, including the kidney [179, 180]. It is detected in all biological fluids in the human body [181]. Physiological concentrations of clusterin in serum range from 35 to 105 *μ*g/ml. However, the concentration is much lower in the cerebrospinal fluid, ranging between 1.2 and 3.6 *μ*g/ml [182]. CLU is found in the tubules with antiapoptotic effects in the kidney and mediates cell protection, lipid recycling, cell attachment, and aggregation [180]. Its increased expression is detected in pathological states [183]. It is involved in many biological processes, including lipid distribution and complement regulation [184, 185]. In addition, the action of clusterin is analogous to the heat shock protein family through its chaperone functions and by helping proteins fold again and adequately after a stressor [186].

Clusterin is a protein whose concentration also increases in kidney disease [187]. Numerous studies have shown that CLU is deposited in the glomeruli as deposits along with complement elements [188]. According to Guo et al. [189], reduced CLU levels negatively affect renal function in ischemia-reperfusion disorders, predisposing to the chronic failure of this organ. Zhou et al. [190] observed that CLU deficiency results in tissue destruction within the kidney and increased cell apoptosis. Kim et al. [191] observed that increase in urine CLU along with albuminuria could be an independent predictive marker for the progression of diabetic kidney disease in type 2 diabetes. CLU reflects the degree of renal tubular damage in the early phase of the disease. These results were confirmed in Zeng et al.'s [192] study, demonstrating that urinary CLU determination can distinguish diabetic nephropathy from albuminuria in patients with type 2 diabetes. Schlatzer et al. [193] believe that CLU can also serve as a good marker in diagnosing type 1 diabetes. On the other hand, according to Solichova et al. [194], the determination of CLU in plasma and serum does not introduce significant changes to the standard routine diagnosis of proteinuria in kidney disease—in studies on clusterin, creatinine, and total protein, no advantage was found for any of the parameters over the others. According to Wu et al. [195], urinary clusterin may be a helpful noninvasive marker in diagnosing kidney damage predisposing to end-stage organ failure in children with systemic lupus erythematosus. In a study in a group of 27 children undergoing allogeneic stem cell transplantation, Musial et al. [196] demonstrated that CLU may be a marker of sublethal renal injury.

The role of CLU in the context of renal transplant rejection has not yet been thoroughly analysed. Only Pianta et al.'s [47] prospective cohort study of renal transplant recipients (*n* = 81) found that CLU may be an essential biomarker of this mechanism when delayed transplant function occurs, with levels increasing as early as 4 hours after surgery.

## 3. Immunosuppression

Immunosuppressive treatment is given to all kidney transplant patients to weaken the immune system so that it does not attack the transplanted organ and cause organ rejection [197]. Currently used immunosuppressive drugs are used in a triple regimen. Calcineurin inhibitors (cyclosporine, tacrolimus), antiproliferative drugs (azathioprine, mycophenolate mofetil), and corticosteroids (prednisone) are used [198]. Other substances that could weaken the immune system have also been investigated in recent years, although not all undergo clinical trials due to harmful side effects [199, 200]. Long-term use of immunosuppressive drugs can have toxic effects on many organs and functions in the human body and the fetus [201, 202].

Immunosuppressive drugs can also affect biomarker levels. Kedzierska et al. [203], in an animal model study, showed that the use of cyclosporine A in rats could increase serum KIM-1 levels. In rats with cyclosporine-induced nephrotoxicity, Hong et al. [204] confirmed higher levels of KIM-1. In a study in children (*n* = 18), Wasilewska et al. [205] noted that both sNGAL and uNGAL significantly increased nephrotoxicity complicated by CsA use.

## 4. Summary

The literature review presented here suggests that NGAL, KIM-1, CXCL-10, CysC, OPN, and CLU may become essential markers in predicting allogeneic kidney transplant rejection. Although not all of them have been thoroughly studied in the context of expression on transplant rejection, they play an essential role in detecting deteriorating renal function. Currently, these biomarkers may have an adjunctive role in the diagnosis of renal rejection alongside standard biochemical parameters and biopsy due to the high sensitivity and specificity and low invasiveness of the assay.

## Figures and Tables

**Table 1:** The concentrations of neutrophil gelatinase-associated lipocalin (NGAL), kidney injury molecule-1 (KIM-1), C-X-C motif chemokine 10 (CXCL-10), cystatin C (CysC), and osteopontin (OPN) in serum and urine (*n*: number of samples; DGF: delayed graft function; IGF: insulin-like growth factors; AKI: acute kidney injury; ARF: acute renal failure; AR: acute rejection; CAD: chronic kidney disease; ACR: biopsy-proven acute cellular rejection; non-R: biopsy-proved nonrejection), ^∗^error in article, should be 338.0 ± 147.2.

Place	*n*	Average age		Concentration of					References
		Donor	Recipient						
NGAL									
Valencia, Spain	38, including 23 (non-DGF) and 15 (DGF)	50.0 ± 20.0 (all patients) 43.0 ± 14.0 (non-DGF) 54.0 ± 12.0 (DGF)	52.0 ± 13.0 (all patients) 49.0 ± 12.0 (non-DGF) 53.0 ± 12.0 (DGF)	In urine (ng/ml)					[21]
				Time	Non-DGF		DGF		
				1 day	92.0		275.0		
				3 days	56.0		258.0		
				6 days	27.0		332.0		
				10 days	21.0		289.0		
Bologna, Italy	43, including 18 (DGF) and 25 (IGF)	52.0 ± 7.9 (all patients) 53.2 ± 8.1 (DGF) 49.9 ± 12.8 (IGF)	54.0 ± 9.6 (all patients) 54.0 ± 8.5 (DGF) 54.0 ± 10.5 (IGF)	In urine (pg/ml)					[22]
				Time	DGF		IGF		
				The day before transplantation	380.7		684.2		
				1 day after transplantation	594.2		289.2		
				3 days after transplantation	491.1		107.6		
				7 days after transplantation	227.8		63.8		
				14 days after transplantation	105.6		33.4		
				30 days after transplantation	31.6		55.7		
Palermo, Italy	29, including 7 (non-DGF) and 22 (DGF)	47.3 ± 20.8 (all patients) 28.4 ± 12.9 (non-DGF) 63.8 ± 7.7 (DGF)	45.2 ± 18.3 (all patients) 58.7 ± 7.8 (non-DGF) 63.8 ± 7.7 (DGF)	In serum (ng/ml)					[23]
				Time	Non-DGF		DGF		
				1 day after transplantation	287.8 ± 162.2		520.7 ± 318.0		
				In urine (ng/ml)					
				Time	Non-DGF		DGF		
				1 day after transplantation	135.8 ± 93.4		47.4 ± 40.3		
Tübingen, Germany	182, including 138 (AR-), 9 (AR+), and 45 (another reason for AKI)		51.0 (AR-) 48.0 (AR+) 58.0 (another reason for AKI)	In urine (ng/ml)					[24]
				Time	AR-	AR+	Another reason of AKI		
				After transplantation	7.8	59.1	339.0		
Tabriz, Iran	37		34.9 ± 15.0	In serum (ng/ml)					[25]
				Time	ARF		Non-ARF		
				Before transplantation	333.6 ± 116.3		300.4 ± 96.2		
				6 hours after transplantation	38.3 ± 147.16^∗^		286.3 ± 66.0		
				12 hours after transplantation	437.3 ± 164.2		252.1 ± 57.5		
Teheran, Iran	27	—	11.2 ± 2.8	In serum (ng/ml)					[26]
				1 day after transplantation	81.2				
				3 days after transplantation	68.0				
				7 days after transplantation	59.1				
				In urine (ng/ml)					
				1 day after transplantation	40.4				
				3 days after transplantation	45.0				
				7 days after transplantation	22.5				
Tokyo, Japan	71, including 12 (AR+) and 59 (AR -)	—	46.6 ± 14.1	In serum (ng/ml)					[27]
				Time	AR+		AR-		
				1 day after transplantation	242.2 ± 125.4		148.4 ± 61.7		
				2 days after transplantation	187.1 ± 83.8		131.1 ± 51.9		
				3 days after transplantation	181.0 ± 75.1		116.5 ± 45.0		
				In urine (ng/ml)					
				Time	AR+		AR-		
				1 day after transplantation	302.8 ± 213.3		130.1 ± 115.5		
				2 days after transplantation	226.4 ± 163.3		66.7 ± 60.0		
				3 days after transplantation	133.2 ± 97.1		46.4 ± 40.2		

KIM-1									
Teheran, Iran	85, including 24 (AR), 19 (CAD), and 42 (no change)		36.8 ± 13.5 (AR) 36.8 ± 13.5 (CAD) 42.0 ± 41.9 (no change)	In serum (in ng/ml)					
				Time	AR	CAD	No change	Control	[28]
				After transplantation	6.7 ± 2.1	8.0 ± 2.3	3.1 ± 1.1	1.5 ± 0.5	
				In urine (ng/mg creatinine)					
				After transplantation	1.9 ± 0.8	2.5 ± 0.7	1.1 ± 0.4	0.6 ± 0.4	

CXCL-10									
Istanbul, Turkey	85, including 70 (non-AR) and 15 (AR)	—	35.9 ± 13.6 (non-AR) 33.4 ± 7.6 (AR)	In urine (ng/ml)					[29]
				Time	Non-AR		AR		
				Before	59.0 ± 8.9		64.0 ± 10.9		
				1 day after transplantation	65.1 ± 24.5		168.9 ± 60.0		
				7 days after transplantation	71.0 ± 25.0		191.5 ± 41.6		
				1 month after transplantation	61.9 ± 13.6		136.2 ± 67.3		
				3 months after transplantation	62.1 ± 9.5		69.2 ± 8.4		
				At the time of rejection	64.3 ± 10.2		242.3 ± 59.4		
				After implementation of antirejection treatment	62.2 ± 11.2		89.1 ± 9.7		

CysC									
Urmia, Iran	49	—	41.2 ± 13.3	In serum (ng/ml)					[30]
				3 days after transplantation	4722.3 ± 2707.6				
				8 days after transplantation	4313.7 ± 2566.7				
				14 days after transplantation	4391.0 ± 2476.2				

OPN									
Shanghai, China	38, including 22 (ACR) and 16 (non-R)		44.0 ± 14.4 (ACR) 45.1 ± 17.4 (non-R)	In plasma (ng/ml)					[31]
					ACR		Non-R		
				After transplantation	41.8 ± 18.5		19.4 ± 8.2		
				In urine (ng/ml)					
				After transplantation	179.5 ± 60.2		98.5 ± 10.3		

**Table 2:** Biomarkers and their main features (DGF: delayed graft function; EGF: epidermal growth factors; SGF: slow graft function; AKI: acute kidney injury; AR: acute rejection; CAD: chronic kidney disease; ABMR: antibody-mediated rejection; CAN: chronic allograft nephropathy; GvHD: graft-versus-host disease).

Biomarker	Sample type	Main features	References
Neutrophil gelatinase-associated lipocalin (NGAL)	—	It predicts AR	[20]
	Urine	It predicts DGF	[21]
	Urine	It predicts DGF	[22]
	Urine	It predicts DGF and chronic allograft nephropathy progression	[23]
	Urine	It predicts AR	[24]
	Plasma	It predicts AKI and graft rejection during the first week after transplantation	[25]
	Urine	It predicts AR	[27]
	Plasma	It predicts DGF	[35]
	Urine	It predicts EGF, DGF, and SGF	[36]
	Urine	It predicts AKI after transplantation	[37]
	Urine	It predicts the change in kidney transplant function	[38]
Kidney injury molecule-1(KIM-1)	Serum and urine	It predicts AR and CAD	[28]
	Serum	It predicts AR	[39]
	Urine	It predicts long-term graft loss	[40]
C-X-C motif chemokine 10 (CXCL-10)	Urine	It predicts ABMR	[10]
	Urine	It predicts T cell-mediated rejection in early posttransplantation period	[29]
	Urine	It predicts AR	[41]
	Serum	It predicts high risk of severe rejection and transplant failure	[42]
	Serum	It predicts AR and CAN	[43]
	Urine	It predicts AR	[44]
Cystatin C (CysC)	Serum	It predicts reduction in kidney function	[45]
Osteopontin (OPN)	Serum	It predicts ACR	[31]
	Cell lines	It predicts GvHD	[46]
Clusterin (CLU)	Urine	It predicts DGF	[47]

**Table 3:** Pros and cons of biomarkers of allogeneic kidney transplant rejection from a clinical perspective.

Biomarkers	Pros	Cons
NGAL	Correlation between high uNGAL concentration and elevated albumin/creatinine ratio [21] Measurement of cumulative NGAL concentrations 1 month after transplantation may predict a weak GFR after 2 years of follow-up [22] uNGAL distinguishes acute allograft rejection from other causes of AKI [24] Serum NGAL may be a predictor of renal rejection if detected as early as 1 day after transplantation [20] An appropriate cutoff value for serum NGAL can distinguish patients with AR from patients with other causes of acute allograft function [27]	In the first hour after transplant surgery, as a result of a large amount of urine excretion, uNGAL levels may be underestimated due to dilution of the urine [23] Induction of NGAL by certain drugs such as cephalosporin, cisplatin, and bisphosphonate [26]
KIM-1	High levels of KIM-1 in serum and urine are inversely related to GFR levels [28] High urinary KIM-1 excretion is a predictor of graft loss, independent of donor age, creatinine clearance, and proteinuria [40]	Renoprotective interventions in kidney injury can inhibit KIM-1 expression [40]
CXCL-10	CXCL-10 levels are significantly higher in individuals with T cell-mediated rejection compared to individuals with antibody-mediated rejection [10, 29] Mean CXCL-10 levels after kidney transplantation may be a predictor of impaired graft function even in the absence of acute rejection [41] CXCL-10 is a more sensitive and predictive parameter than serum creatinine in terms of monitoring response to antirejection therapy [42]	CXCL-10 concentration is not useful for determining DGF [29]
CysC	Serum cystatin C in case of GFR loss is a better marker than creatinine [45] Cystatin C has a significantly higher sensitivity than serum creatinine in its ability to detect a decrease in GFR < 60 ml/min in renal transplant recipients [45]	The strength of the correlation of cystatin C with renal rejection is strongly dependent on the timing of CysC determination after transplantation [30]
OPN	Plasma OPN levels were positively correlated with the severity of biopsy-proven acute cellular rejection [31]	OPN is probably a nonsignificant regulator of apoptosis in acute rejection [48]
CLU	CLU in plasma may be a significant biomarker of DGF as early as 4 hours after kidney transplantation [47]	The lack of rapid tests for clusterin hinders rapid clinical application, although rapid tests are available for many proteins, including NGAL and KIM-1 [47]
